# Supplementary material for: Association between the blaCTX-M-14-harboring Escherichia coli Isolated from Weasels and Domestic Animals Reared on a University Campus
Source: Antibiotics (Basel). 2021 Apr 13;10(4):432. doi: 10.3390/antibiotics10040432 (PMC8069031; doi:10.3390/antibiotics10040432)
Supplement: Supplementary file 1 [file antibiotics-10-00432-s001.pdf]

Table S1. Average nucleotide identity (%) and aligned nucleotides (%, number of aligned nucleotides between query and reference sequences/bases in length of reference sequence) of six plasmid sequences.

**Query**<br>**Reference**	**p105CF**<br>**(99,001 bp)**	**p130MS**<br>**(98,904 bp)**	**p74DF1**<br>**(99,262 bp)**	**p80DF1**<br>**(98,904 bp)**	**p116DF2**<br>**(99,067 bp)**	**p123DF2**<br>**(105,498 bp)**
p105CF<br>(99,001 bp)	*	(100)<br>[98.07]<br>97089/99001	(100)<br>[97.82]<br>96847/99001	(99.99)<br>[98.43]<br>97443/99001	(100)<br>[97.05]<br>96076/99001	(100)<br>[94.94]<br>93992/99001
p130MS<br>(98,904 bp)	(100)<br>[97.83]<br>96753/98904	*	(100)<br>[97.37]<br>96304/98904	(100)<br>[97.83]<br>96753/98904	(100)<br>[97.60]<br>96530/98904	(100)<br>[95.92]<br>94870/98904
p74DF1<br>(99,262 bp)	(100)<br>[97.59]<br>96870/99262	(99.98)<br>[97.74]<br>97023/99262	*	(99.98)<br>[97.74]<br>97021/99262	(100)<br>[97.47]<br>96754/99262	(99.99)<br>[97.02]<br>96305/99262
p80DF1<br>(98,904 bp)	(100)<br>[97.87]<br>96799/98904	(100)<br>[97.83]<br>96762/98904	(99.98)<br>[98.03]<br>96956/98904	*	(100)<br>[97.57]<br>96496/98904	(100)<br>[95.94]<br>94884/98904
p116DF2<br>(99,067 bp)	(100)<br>[97.80]<br>96888/99067	(99.98)<br>[97.84]<br>96931/99067	(99.98)<br>[96.94]<br>96035/99067	(100)<br>[97.80]<br>96888/99067	*	(99.98)<br>[95.64]<br>94747/99067
p123DF2<br>(105,498 bp)	(100)<br>[89.10]<br>94000/105498	(100)<br>[89.97]<br>94917/105498	(99.99)<br>[89.43]<br>94349/105498	(100)<br>[89.32]<br>94227/105498	(99.98)<br>[90.97]<br>95968/105498	*

* Homologous sequence.
